# Supplementary material for: Autofluorescence Virtual Staining System for H&E Histology and Multiplex Immunofluorescence Applied to Immuno-Oncology Biomarkers in Lung Cancer
Source: Cancer Res Commun. 2025 Jan 8;5(1):54–65. doi: 10.1158/2767-9764.CRC-24-0327 (PMC11707747; doi:10.1158/2767-9764.CRC-24-0327)
Supplement: Supplementary Material 1 [file crc-24-0327_supplementary_material_1_suppsm1.pdf]

Supplementary Material 1

Pseudo IHC

To render the corresponding pIHC image from the mIF image, **Equation S1** was applied to all pixels in the image. Note that **Equation S1** is invertible and can also be used to render the mIF image from the corresponding pIHC image.

$$\begin{bmatrix} IHC_{\mathrm{r}} \\ IHC_{\mathrm{g}} \\ IHC_{\mathrm{b}} \end{bmatrix} = \begin{bmatrix} B_{\mathrm{r}} \\ B_{\mathrm{g}} \\ B_{\mathrm{b}} \end{bmatrix} + \left(\begin{bmatrix} W_{\mathrm{r}} \\ W_{\mathrm{g}} \\ W_{\mathrm{b}} \end{bmatrix} - \begin{bmatrix} B_{\mathrm{r}} \\ B_{\mathrm{g}} \\ B_{\mathrm{b}} \end{bmatrix} \right) \exp\left(- \begin{bmatrix} \alpha_{\mathrm{dapi,r}} & \alpha_{\mathrm{target,r}} & \alpha_{\mathrm{residual,r}} \\ \alpha_{\mathrm{dapi,g}} & \alpha_{\mathrm{target,g}} & \alpha_{\mathrm{residual,g}} \\ \alpha_{\mathrm{dapi,b}} & \alpha_{\mathrm{target,b}} & \alpha_{\mathrm{residual,b}} \end{bmatrix} \begin{bmatrix} IF_{\mathrm{dapi}} \\ IF_{\mathrm{target}} \\ IF_{\mathrm{residual}} \end{bmatrix} \right) \text{(Equation S1)}$$

where

$$\begin{aligned} \mathrm{r, g, b} &= \text{The red, green, and blue channels} \\ B &= \text{The black offset color} \\ W &= \text{The white offset color} \\ \alpha &= \text{The absorption coefficient} \\ IHC &= \text{The IHC pixel value} \\ IF &= \text{The IF pixel value} \end{aligned}$$

To avoid oversaturation of the tissue background, IF_{residual} was clipped to a maximum value of 20. As a result, only IF_{dapi} and IF_{target} can be fully recovered from the inverse transformation.

The offset colors and absorption coefficients are specific to the imaging system and staining protocols used, and were empirically determined based on several factors such as visual quality and similarity to real IHC in-house reference images. **Supplementary Table S1** shows the values used for different targets.
