# Supplementary material for: Autofluorescence Virtual Staining System for H&E Histology and Multiplex Immunofluorescence Applied to Immuno-Oncology Biomarkers in Lung Cancer
Source: Cancer Res Commun. 2025 Jan 8;5(1):54–65. doi: 10.1158/2767-9764.CRC-24-0327 (PMC11707747; doi:10.1158/2767-9764.CRC-24-0327)
Supplement: Supplementary Material 2 [file crc-24-0327_supplementary_material_2_suppsm2.pdf]

Supplementary Material 2

Hyperparameters

Supplementary Table S2 shows the hyperparameter details for the H&E and mIF virtual stainer models.

The learning rate schedule consisted of a linear warmup and cosine decay. Specifically, the learning rate was increased linearly over a certain number of warmup training steps, and decreased at a fixed decay rate with cosine annealing for the remaining training steps.

The loss schedule changed the relative weights of the shift-invariant regression loss and adversarial loss components over time. Specifically, the L1 and L2 regression weights of the shift-invariant regression loss decreased linearly from the initial weight to final weight over the training steps, whereas the conditional and unconditional GAN weights of the adversarial loss increased linearly. If the loss schedule was not employed, the initial and final weights remained the same.
