# Supplementary material for: Autofluorescence Virtual Staining System for H&E Histology and Multiplex Immunofluorescence Applied to Immuno-Oncology Biomarkers in Lung Cancer
Source: Cancer Res Commun. 2025 Jan 8;5(1):54–65. doi: 10.1158/2767-9764.CRC-24-0327 (PMC11707747; doi:10.1158/2767-9764.CRC-24-0327)
Supplement: Supplementary Material 3 [file crc-24-0327_supplementary_material_3_suppsm3.pdf]

Supplementary Material 3

mIF Evaluation Methods

For mIF stains, measurements of the positive cell density, positive cell percentage, TPS, and CPS were calculated based on **Equations S2 - S5**.

$$\text{Positive cell count} = \text{Number of positive cells within region}$$

$$\text{Positive cell density} = \frac{\text{Positive cell count}}{\text{Area of region}} \qquad \text{(Equation S2)}$$

$$\text{Positive cell percentage} = 100 \times \frac{\text{Positive cell count}}{\text{Number of cells within region}} \qquad \text{(Equation S3)}$$

$$\text{TPS} = 100 \times \frac{\text{Number of PD-L1 positive cells within tumor region}}{\text{Number of cells within tumor region}} \qquad \text{(Equation S4)}$$

$$\text{CPS} = 100 \times \frac{\text{Number of PD-L1 positive cells within tissue region}}{\text{Number of cells within tumor region}} \qquad \text{(Equation S5)}$$
