# Supplementary material for: Autofluorescence Virtual Staining System for H&E Histology and Multiplex Immunofluorescence Applied to Immuno-Oncology Biomarkers in Lung Cancer
Source: Cancer Res Commun. 2025 Jan 8;5(1):54–65. doi: 10.1158/2767-9764.CRC-24-0327 (PMC11707747; doi:10.1158/2767-9764.CRC-24-0327)
Supplement: Supplementary Material 4 [file crc-24-0327_supplementary_material_4_suppsm4.pdf]

# Supplementary Material 4

## mIF Evaluation Results

### Qualitative Analysis

**Supplementary Figure S1** shows examples of real and virtual stains of various morphological structures at various magnifications from each individual model. **Supplementary Figure S2** shows examples of false negatives or false positives in the virtual stains. **Supplementary Figure S3** shows examples of background and non-specific fluorescence observed in the CD8 real stains which adds label noise during the model training procedure, resulting in poorer performance.

### Quantitative Analysis

**Supplementary Tables S3** and **S4** show the average absolute differences between the measurements on real and virtual stains obtained from the cell segmentation-based analysis in Visiopharm software for the single expression and colocalization analysis, respectively. Blank entries indicate measurements that were not relevant for the stain as described in **Materials and Methods**. **Supplementary Figures S4 - S11** show the scatterplots of measurements on real and virtual stains.
