# Supplementary material for: Autofluorescence Virtual Staining System for H&E Histology and Multiplex Immunofluorescence Applied to Immuno-Oncology Biomarkers in Lung Cancer
Source: Cancer Res Commun. 2025 Jan 8;5(1):54–65. doi: 10.1158/2767-9764.CRC-24-0327 (PMC11707747; doi:10.1158/2767-9764.CRC-24-0327)
Supplement: Supplementary Table S1 [file crc-24-0327_supplementary_table_s1_suppst1.pdf]

Supplementary Table S1: Values used for different targets in the pIHC algorithm.

Value	PanCK	PD-L1	CD3	CD8
B_r	50	50	50	50
B_g	50	50	50	50
B_b	50	50	50	50
W_r	228	228	228	228
W_g	225	225	225	225
W_b	233	233	233	233
$\alpha_{dapi,r}$	0.008	0.00459	0.004	0.004
$\alpha_{dapi,g}$	0.006872	0.003174	0.003436	0.003436
$\alpha_{dapi,b}$	0.003376	0.001056	0.001688	0.001688
$\alpha_{target,r}$	0.009	0.01119	0.009	0.009
$\alpha_{target,g}$	0.01992	0.02058	0.01992	0.01992
$\alpha_{target,b}$	0.03	0.0294	0.03	0.03
$\alpha_{residual,r}$	0.0021	0.0021	0.0021	0.0021
$\alpha_{residual,g}$	0.00255	0.00255	0.00255	0.00255
$\alpha_{residual,b}$	0.003	0.003	0.003	0.003
