# Supplementary material for: Autofluorescence Virtual Staining System for H&E Histology and Multiplex Immunofluorescence Applied to Immuno-Oncology Biomarkers in Lung Cancer
Source: Cancer Res Commun. 2025 Jan 8;5(1):54–65. doi: 10.1158/2767-9764.CRC-24-0327 (PMC11707747; doi:10.1158/2767-9764.CRC-24-0327)
Supplement: Supplementary Table S2 [file crc-24-0327_supplementary_table_s2_suppst2.pdf]

Supplementary Table S2: Hyperparameter values used for the H&E and mIF virtual stainer models.

Component	Hyperparameter	H&E	mIF
Generator	Kernel size	3×3	3×3
	Number of kernels	384, 768, 1536, 3072	128, 256, 512, 1024
	Dropout	0.3	0.3
	Attention gate	False	True
Conditional discriminator	Kernel size	4×4	4×4
	Number of kernels	384, 768, 1536, 3072	128, 256, 512, 1024
	Dropout	0.1	0.1
Unconditional discriminators	Kernel size	4×4	4×4
	Number of kernels	384, 768, 1536, 3072	128, 256, 512, 1024
	Dropout	0.1	0.1
Loss	Schedule	True	False
	L1 regression weight (Initial, Final)	95, 30	95, 95
	L2 regression weight (Initial, Final)	5, 5	5, 5
	Conditional GAN weight (Initial, Final)	0.1, 10	100, 100
	Unconditional GAN1 weight (Initial, Final)	0.07, 7	1, 1
	Unconditional GAN2 weight (Initial, Final)	0.025, 2.5	10, 10
	Rotational consistency weight	100	100
	Regularization weight	0	0.001

Optimizer	Schedule	True	True
	Learning rate	5e-5	5e-5
	Warmup steps	10240	10240
	Decay rate	0.9	0.9
	$\boldsymbol{\beta}_1$	0.5	0.5
	$\boldsymbol{\beta}_2$	0.999	0.999
Training	Batch size	16	32
