# Supplementary material for: Autofluorescence Virtual Staining System for H&E Histology and Multiplex Immunofluorescence Applied to Immuno-Oncology Biomarkers in Lung Cancer
Source: Cancer Res Commun. 2025 Jan 8;5(1):54–65. doi: 10.1158/2767-9764.CRC-24-0327 (PMC11707747; doi:10.1158/2767-9764.CRC-24-0327)
Supplement: Supplementary Table S3 [file crc-24-0327_supplementary_table_s3_suppst3.pdf]

Supplementary Table S3: Average absolute difference (mean ± SD, median) between measurements on real and virtual stains obtained from the cell segmentation-based analysis in Visiopharm software for PanCK, DAPI, PD-L1, CD3, and CD8 on testing slides. Analysis was performed according to three different definitions of the region of interest.

Region	Measurement	PanCK	DAPI	PD-L1	CD3	CD8
Tissue	Positive area (mm^2)	6.7 ± 6.5, 4.7	-	-	-	-
	Positive cell density (cells/mm^2)	-	306 ± 388, 201	435 ± 357, 340	162 ± 170, 111	169 ± 188, 104
	Positive cell percentage (%)	-	-	10.5 ± 6.9, 9.3	3.6 ± 3.2, 2.7	4.1 ± 4.4, 2.7
Real tumor	Positive cell density (cells/mm^2)	-	557 ± 672, 354	820 ± 782, 630	205 ± 194, 169	180 ± 227, 100
	Positive cell percentage (%)	-	-	See TPS and CPS	2.9 ± 2.6, 2.2	2.8 ± 3.5, 1.7
	TPS (%)	-	-	11.9 ± 8.6, 11.5	-	-
	CPS (%)	-	-	18.1 ± 12.1, 16.3	-	-
Respective tumor	Positive cell density (cells/mm^2)	-	845 ± 778, 674	834 ± 821, 608	278 ± 260, 200	192 ± 236, 110
	Positive cell percentage (%)	-	-	See TPS and CPS	3.6 ± 3.0, 3.0	2.8 ± 3.6, 1.7
	TPS (%)	-	-	11.7 ± 8.7, 11.5	-	-
	CPS (%)	-	-	20.8 ± 15.5, 16.1	-	-
