# Supplementary material for: Autofluorescence Virtual Staining System for H&E Histology and Multiplex Immunofluorescence Applied to Immuno-Oncology Biomarkers in Lung Cancer
Source: Cancer Res Commun. 2025 Jan 8;5(1):54–65. doi: 10.1158/2767-9764.CRC-24-0327 (PMC11707747; doi:10.1158/2767-9764.CRC-24-0327)
Supplement: Supplementary Table S4 [file crc-24-0327_supplementary_table_s4_suppst4.pdf]

Supplementary Table S4: Average absolute difference (mean ± SD, median) between measurements on real and virtual stains obtained from the colocalization analysis in Visiopharm software for CD3 and CD8, and CD3 and PD-L1, on testing slides. Analysis was performed according to three different definitions of the region of interest.

**Region**	**Measurement**	**CD3 and CD8**	**CD3 and PD-L1**
Tissue	Positive cell density (cells/mm^2)	94 ± 95, 65	112 ± 126, 71
	Positive cell percentage (%)	2.2 ± 2.1, 1.5	2.5 ± 2.3, 1.8
Real tumor	Positive cell density (cells/mm^2)	106 ± 118, 64	155 ± 207, 91
	Positive cell percentage (%)	1.5 ± 1.6, 1.0	2.1 ± 2.2, 1.3
Respective tumor	Positive cell density (cells/mm^2)	91 ± 106, 59	170 ± 225, 105
	Positive cell percentage (%)	1.4 ± 1.5, 1.0	2.2 ± 2.2, 1.6
