# Supplementary material for: Autofluorescence Virtual Staining System for H&E Histology and Multiplex Immunofluorescence Applied to Immuno-Oncology Biomarkers in Lung Cancer
Source: Cancer Res Commun. 2025 Jan 8;5(1):54–65. doi: 10.1158/2767-9764.CRC-24-0327 (PMC11707747; doi:10.1158/2767-9764.CRC-24-0327)
Supplement: Supplementary Figure S1 [file crc-24-0327_supplementary_figure_s1_suppsf1.pdf]

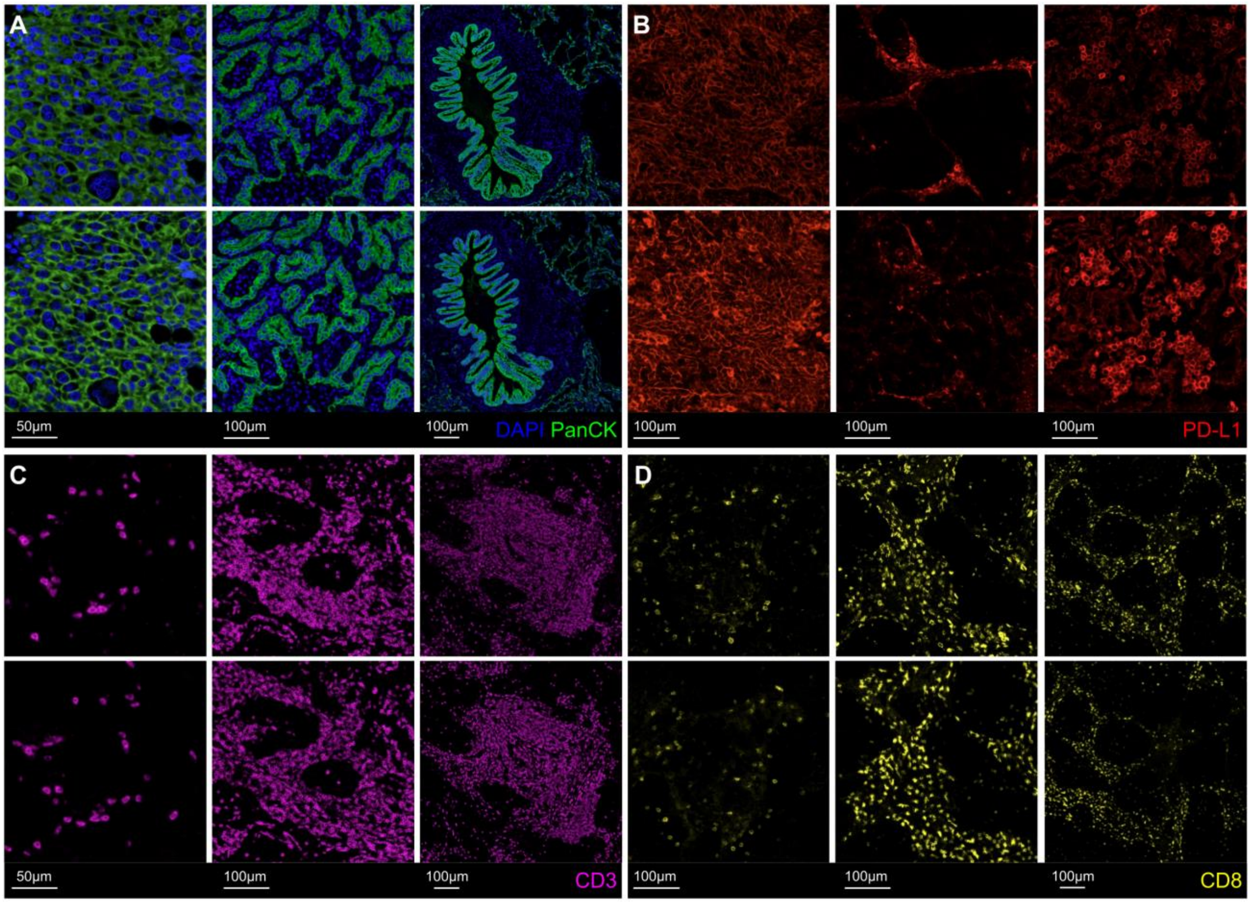

Supplementary Figure S1: (A) Examples of the real (top) and virtual (bottom) stains for DAPI and PanCK showing various morphological features at 40× (left), 20× (middle) and 10× (right) magnifications. **(B)** Examples of the real (top) and virtual (bottom) stains for PD-L1 showing positive expression in tumor cells at 20× magnification (left), negative expression in tumor cells at 20× magnification (middle), and positive expression in immune cells at 20× magnification (right). **(C)** Examples of the real (top) and virtual (bottom) stains for CD3 showing a low density region at 40× magnification (left), high density region at 20× magnification (middle), and tertiary lymphoid structure at 10× magnification (right). **(D)** Examples of the real (top) and virtual (bottom) stains for CD8 showing a low density region at 20× magnification (left), high density region at 20× magnification (middle), and high density region at 10× magnification (right).
