# Supplementary material for: Autofluorescence Virtual Staining System for H&E Histology and Multiplex Immunofluorescence Applied to Immuno-Oncology Biomarkers in Lung Cancer
Source: Cancer Res Commun. 2025 Jan 8;5(1):54–65. doi: 10.1158/2767-9764.CRC-24-0327 (PMC11707747; doi:10.1158/2767-9764.CRC-24-0327)
Supplement: Supplementary Figure S2 [file crc-24-0327_supplementary_figure_s2_suppsf2.pdf]

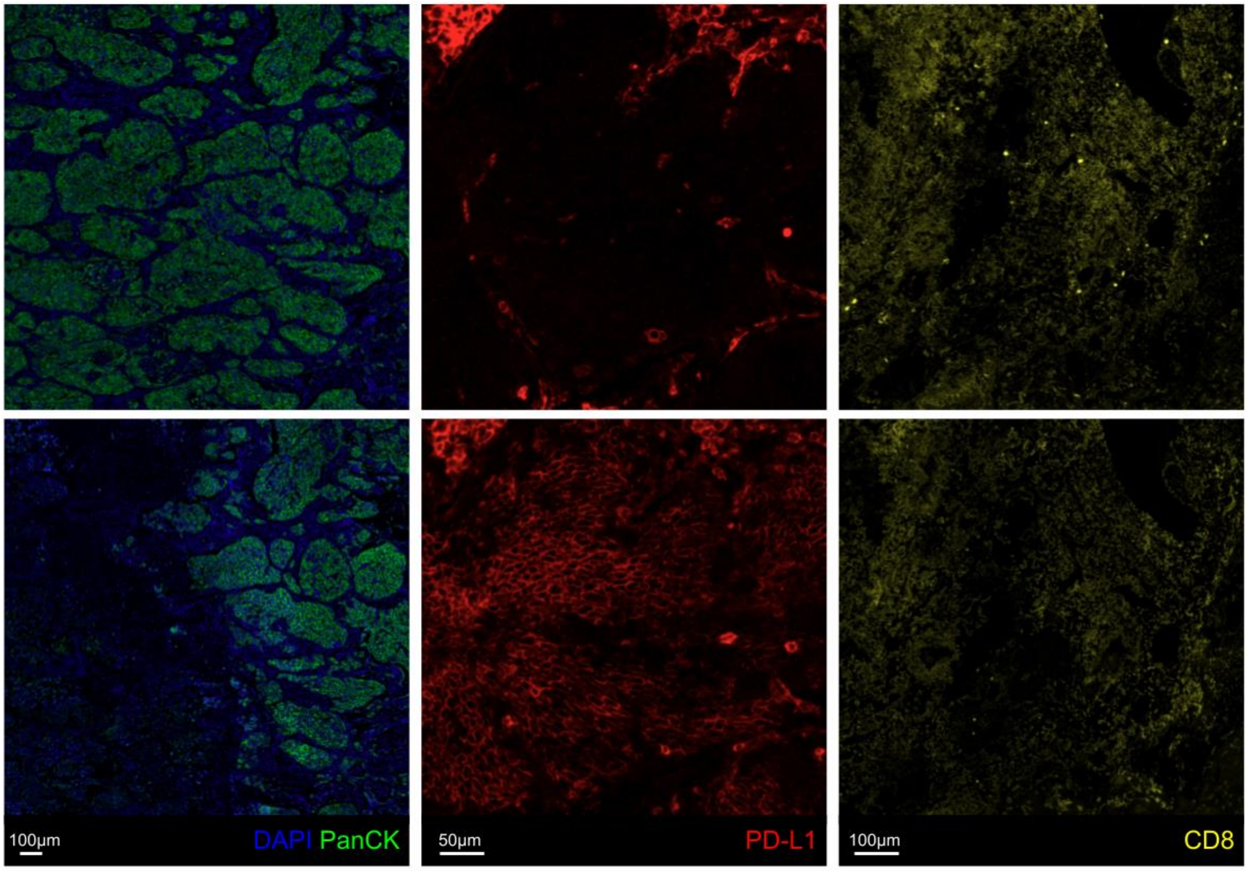

Supplementary Figure S2: Examples of the real (top) and virtual (bottom) stains showing false negative regions for the PanCK virtual stain (left), false positive regions for the PD-L1 virtual stain (middle), and false negative cells for the CD8 virtual stain (right).
