# Supplementary material for: Autofluorescence Virtual Staining System for H&E Histology and Multiplex Immunofluorescence Applied to Immuno-Oncology Biomarkers in Lung Cancer
Source: Cancer Res Commun. 2025 Jan 8;5(1):54–65. doi: 10.1158/2767-9764.CRC-24-0327 (PMC11707747; doi:10.1158/2767-9764.CRC-24-0327)
Supplement: Supplementary Figure S7 [file crc-24-0327_supplementary_figure_s7_suppsf7.pdf]

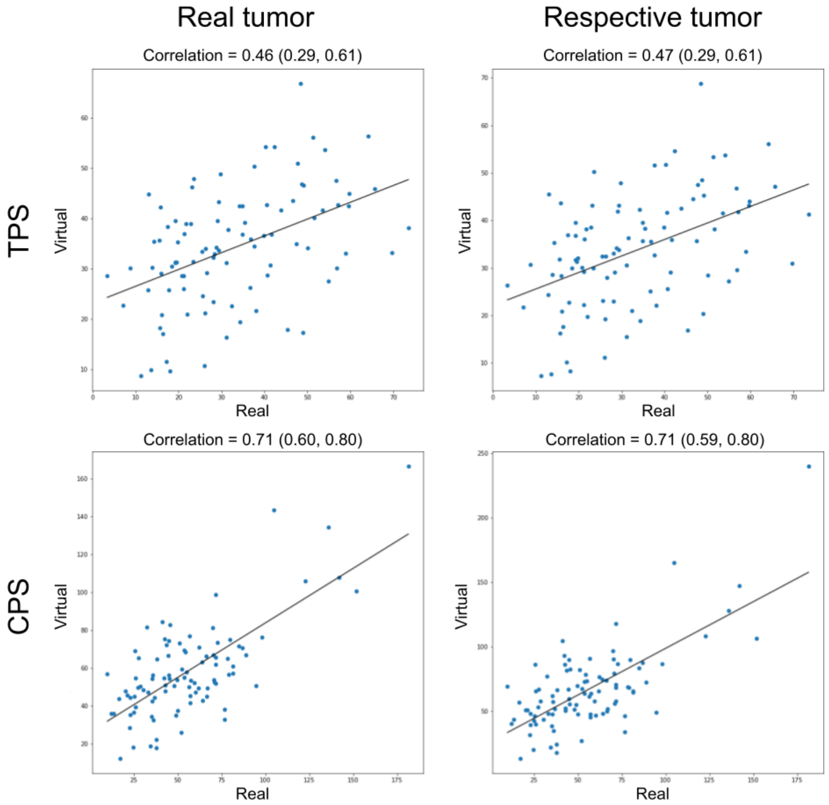

Supplementary Figure S7: Scatterplots of the measurements on real and virtual stains of TPS and CPS for PD-L1.
