# Supplementary material for: Autofluorescence Virtual Staining System for H&E Histology and Multiplex Immunofluorescence Applied to Immuno-Oncology Biomarkers in Lung Cancer
Source: Cancer Res Commun. 2025 Jan 8;5(1):54–65. doi: 10.1158/2767-9764.CRC-24-0327 (PMC11707747; doi:10.1158/2767-9764.CRC-24-0327)
Supplement: Supplementary Figure S10 [file crc-24-0327_supplementary_figure_s10_suppsf10.pdf]

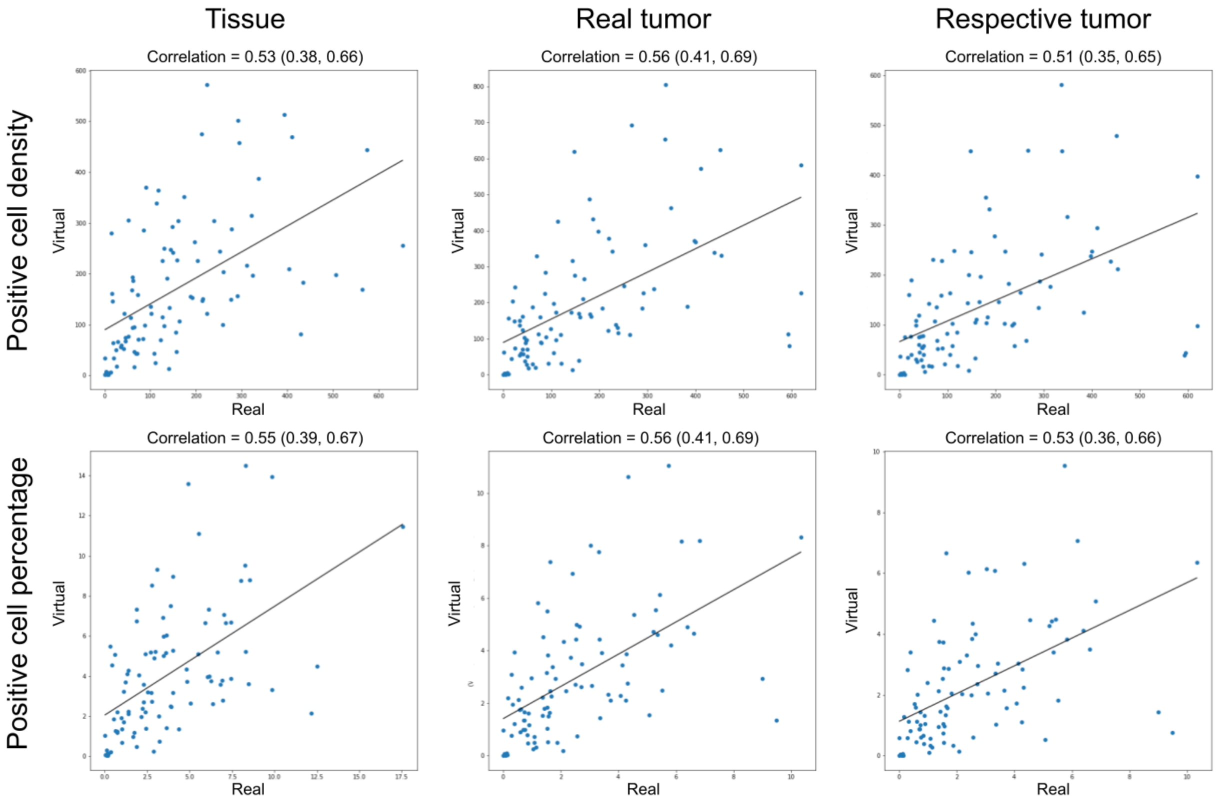

Supplementary Figure S10: Scatterplots of the measurements on real and virtual stains of positive cell density and positive cell percentage for CD3 and CD8 colocalization.
